# Getting local: focusing on communities to achieve greater impact in the next phase of the HIV response

**DOI:** 10.1002/jia2.25366

**Published:** 2019-07-19

**Authors:** Ade O Fakoya, Mark R Dybul, Peter Sands

**Affiliations:** ^1^ The Global Fund to Fight AIDS, Tuberculosis and Malaria Geneva Switzerland; ^2^ Department of Infection and Population Health University College London UK; ^3^ Center for Global Health Practice and Impact Georgetown University School of Medicine Washington USA; ^4^ Joep Lange Institute Amsterdam the Netherlands; ^5^ Harvard Global Health Institute Cambridge USA

**Keywords:** HIV, programme quality, communities, human‐centred design, focused geographies, cities

Despite numerous achievements in addressing HIV epidemics worldwide, there is still much more that needs to be done to control HIV as a public health threat 1. As such, the world is at a critical juncture in the HIV response. Decelerating the response now would reverse the enormous gains achieved so far 2.

Many countries are not on track to reach the ambitious treatment and prevention goals set by the Joint United Nations Programme on HIV/AIDS (UNAIDS) and others. Although several national programmes are making progress when statistics are rolled up to the national level 3, too often at district and local levels, many of the key and vulnerable populations are being left behind. Some groups remain disproportionally affected by HIV and are conspicuously absent or under‐represented in HIV services. Adolescent girls and young women, sex workers, heterosexual men in high HIV prevalence setting, men who have sex with men, transgender people and incarcerated individuals continue to be underserved by HIV prevention and treatment programmes 4. Recently, there has been increased attention on the low uptake of HIV testing and treatment in heterosexual men 5 and the very low percentage of adolescents who know their status. In one study of 16 African countries, men were up to 70% less likely to know their status compared to women. The figure for adolescents aged 16 to 19 years was 40% compared to the 30‐ to 39‐year‐old age group 6.

The rapid scaling up of treatment has led to over 20 million people receiving life‐saving HIV medication; however, the quality of services has been inconsistent across different settings. In some places, a staggering two‐fifths (40%) of people on therapy are lost to follow‐up 7, 8. Additionally, due to variable levels of viral suppression (caused by a shortened duration of anti‐retro‐viral therapy (ART) access and poor ART adherence at the individual level), the number of persons acquiring primary HIV‐resistant virus is increasing 9, 10. Even where coverage of ART is high, the impact on preventing new infections has so far failed to reach levels that had been predicted, and incidence rates remain too high for long‐term epidemic control 11.

Therefore, there is an urgent need for new approaches and tools to tackle stubbornly persistent and alarmingly high incidence rates in the above‐mentioned groups.

As the HIV response becomes increasingly medicalized, engagement of, and funding for, community‐level interventions are threatened. There is little hope of succeeding in reaching the most vulnerable without greater community leadership.

Finally, there is insufficient focus on reducing the cost of delivery, including greater impact for investment. There is a growing body of evidence on how to maximize impact (including through differentiated service delivery) but the disconnect from country‐level decision‐making, community engagement and delivery make significant progress challenging. Significant cost savings could open fiscal space to help ensure key components of an effective response receive more funding 12.

Increasingly, countries are focusing on several critical areas to drive the responses towards greater impact and greater sustainability by aligning services to the identified needs of individuals and communities. Ensuring that there is a fully funded response with efficient utilization of domestic and external resources is fundamental 13. We would suggest four other critical elements: (a) adapting services based on community involvement and demand, so, called human‐centred design; (b) “taking AIDS out of isolation” by addressing the wider determinants of health and social wellbeing including gender norms, stigma and the economic costs of accessing health services, including user fees 2; (c) using data to identify and map where most infections are occurring, and (while respecting equity) targeting high‐quality differentiated, comprehensive prevention and treatment interventions to specific localities; and (d) capacitating communities to work across all levels of the health response, enabling them to utilize and respond to evidence and achieve impact 14.

Involving communities in the design, monitoring and evaluation of services takes time and resources. But tailoring services to individual needs is one aspect of service quality 15 and the Lancet Global Health Commission on High‐Quality Health Systems in the Sustainable Development Goals Era report has recently highlighted patient experience as a key component of quality health services 16. Often individual barriers to optimal engagement and access to care such as mental health problems, substance use, violence and stigma may not immediately be apparent 17. There are many examples within the literature where services have been adapted, or differentiated to tailor to specifically identified needs or issue, from developing community‐based strategies to improve men's access to HIV testing and treatment 18 to tailoring clinic times and environments to improve accessibility for adolescents 19. Although it is early in the process, the human‐centred design methodology has been used to improve several aspects of service delivery, including access to pre‐exposure prophylaxis 20 and services for adolescents 21.

Considering HIV within a wider context has multiple facets. These include integrating HIV into broader health services to improve HIV and non‐HIV‐related health outcomes; exploring the synergies and co‐dependencies of HIV investments on health systems; and examining the benefits of strong health and community systems to reach HIV and other development goals 22.

The focus on critical populations and locations is not new 23, 24, and many countries and cities within countries are adopting this focus 25, 26. Already more than half the world's population live in cities, which are uniquely positioned to lead the response towards achieving the UNAIDS treatment and prevention targets. Cities may also be HIV hotspots, and people living in or near a hotspot have a much higher risk of acquiring HIV infection compared to those living outside of the hotspot 27.

By adding emphasis on delivering services aligned with community‐identified needs, and by linking the outcomes of this human‐centred design process to regional and national policy makers and high‐level decision makers, we can provide a model which has potential to drive a more effective response. Several countries are now exploring these methodologies.

One final aspect of service adaptation to address the needs of particular groups is the issue of scale. It is not enough that services cater for a limited number of clients; impact can only be feasibly achieved if we move from demonstration projects to full coverage at scale. This can be achieved 19, but often it is a critical limitation of implementing these approaches which must be addressed.

Achieving a greater impact in the next phase of the HIV response requires an improvement in the quality and integration of services to make the services align to the identified needs of communities.

## Competing interests

AF and PS report no conflicts of interest.

MD received a research grant from the Bill and Melinda Gates Foundation to support work on human‐centred design.

## Authors’ contributions

All authors contributed equally to this work.
